# Editorial: Nanomaterials to combat pathogenic microorganisms

**DOI:** 10.3389/fmicb.2023.1160196

**Published:** 2023-04-13

**Authors:** Mehran Alavi, Danial Kahrizi, Fleming Martinez, Mahendra Rai

**Affiliations:** ^1^Department of Biological Science, Faculty of Science, University of Kurdistan, Sanandaj, Iran; ^2^Nanobiotechnology Department, Faculty of Innovative Science and Technology, Razi University, Kermanshah, Iran; ^3^Agricultural Biotechnology Department, Faculty of Agriculture, Tarbiat Modares University, Tehran, Iran; ^4^Department of Pharmacy, Faculty of Sciences, Universidad Nacional de Colombia, Bogotá, Colombia; ^5^Nanobiotechnology Laboratory, Department of Biotechnology, Sant Gadge Baba (SGB) Amravati University, Amravati, Maharashtra, India; ^6^Department of Microbiology, Nicolaus Copernicus University, Toruń, Poland

**Keywords:** pathogenic microorganisms, antibiotic-resistant bacteria, nanomaterials, photodynamic inactivation, metal and metal oxide nanoparticles

In the present scenario, there are frightening reports of new and emerging microbial pathogens such as severe acute respiratory syndrome coronavirus 2 (SARS-CoV-2) which has threatened human health globally causing the outbreak of disease. In addition, some studies indicate the development of resistance in pathogenic microbes, for example, *Candida auris* has become a global threat evidenced by high mortality due to the development of resistance to different antimicrobials. This has generated urgent attention of the scientific community to discover a new class of antimicrobials that are effective against new, emerging, and resistant microbes.

In recent years, nanotechnology has offered novel strategies for the inhibition of new, emerging, or resistant microbial pathogens based on synthetic, natural, and engineered nanomaterials (Choi, 2021; Jacobs et al., 2022). There is a wide range of inorganic, organic, and carbon-based nanomaterials (Adefegha et al., 2022). The nanomaterials are considered non-traditional antimicrobial materials that are effective and alternative to traditionally used antimicrobials. These are the new tools and frontline of materials to combat drug-resistant pathogens (Garg et al., 2022).

Comparing the efficiency of each antimicrobial agent *in vitro* and *in vivo* is a critical issue, particularly in the case of health-threatening microorganisms such as multidrug-resistant bacteria, fungi, and viruses (Alavi et al., 2022). For instance, antibiotic-resistant bacteria can neutralize an antibiotic by production of a specific enzyme, overexpression of efflux pumps, and modification of a drug target as the major mechanisms of antibiotic resistance (Amraei et al., 2022).

Nanomaterials can be synthesized by physical, chemical, and biological techniques. The latter is eco-friendly, easy, and economically viable that can be applied at ambient temperature and pressure. Among the nanomaterials, silver nanoparticles have been researched extensively due to their potential antimicrobial activity and are thus considered a new generation of antimicrobials (Rai et al., 2009) Biosynthesized nanoparticles are biocompatible and are known for better bioactivities. The paper contributed by Jang et al. discussed the biosynthesis of silver nanoparticles (AgNPs) by using an extract of *Viola betonicifolia* and evaluated antimicrobial activity against *Helicobacter pylori, Staphylococcus epidermidis, Candida tropicalis*, and *Trichophyton rubrum*. In addition, the authors assessed the antibiofilm activity of the above-mentioned pathogens. Further, they reported superior antimicrobial /antibiofilm and cytobiocompatibility. In another promising study, Bernardo et al. synthesized AgNPs by *Syzygium cumini* leaf extract. They also reported excellent bioactivities (antimicrobial, antibiofilm, and cytotoxicity) against *Actinomyces naeslundii, Fusobacterium nucleatum, Staphylococcus aureus, Staphylococcus epidermidis, Streptococcus mutans, Streptococcus oralis, Veillonella dispar*, and *Candida albicans*. The cytotoxicity was found to be time and dose-dependent. Similarly, Wei et al. in their study used extract of *Mahonia fortunei* for the synthesis of AgNPs and assessed their efficacy against *Pectobacterium carotovorum*, a bacterium that causes soft rot of cabbage, and other medicinally important bacteria *Staphylococcus aureus, Escherichia coli, Bacillus subtilis*, and *Pseudomonas aeruginosa*. Interestingly, 8 μg ml^−1^ showed remarkable results by inhibiting the bacteria completely. Likewise, it was found that the bacterial membrane deteriorated and biofilm formation was inhibited. Further, AgNPs demonstrated synergistic activity against *Pectobacterium carotovorum* when used in combination with zhongshengmycin. The authors concluded that the AgNPs synthesized by *Mahonia fortunei* can be applied against *Pectobacterium carotovorum*.

The phytosynthesized nanoparticles have garnered the attention of nanotechnologists and are now well-known for their wide range of bioactivities. In a comprehensive review, Abolarinwa et al. discussed efficacy of plant-derived nanoparticles against bacterial pathogens causing diarrhea such as *Vibrio cholerae, Escherichia coli, Shigella, Salmonella, Campylobacter*, and *Clostridium* species that have developed resistance and are now known as the global health crisis, Such bacteria can be treated with plant-derived nanoparticles as an alternative green strategy.

Mycosynthesis is another method of synthesis of nanomaterials. In a study, Gaikwad et al. mycosynthesized AgNPs by using *Fusarium oxysporum* and developed a nanogel that has demonstrated excellent wound healing activity in rats at different concentrations (0.1, 0.5, and 1.0 mg g^−1).^ The nanogel thus developed showed remarkable efficiency of wound healing in excision, incision, and burn wound-healing models, and can be used for wound dressing and healing.

In an interesting study by Zhang and Lo polymer functionalized AgNPs were evaluated for their physicochemical and biological properties. The antimicrobial activity was assessed against *Porphyromonas gingivalis* and found that the polymer functionalization does not change the physicochemical properties such as size and surface charge, however, the surface chemical property was altered in the biological property. These changes lead to significant antibacterial and antibiofilm activities.

Ye et al. argued that due to the rise of multidrug-resistant bacteria like methicillin-resistant *Staphylococcus aureus* (MRSA), the treatment of such infections is an arduous task. To treat this infection, they synthesized copper-containing ferrite nanoparticles (Cu@Fe NPs) which completely retained oxidation-reduction activity. These nanoparticles demonstrated an exceptional drug safety profile and inhibited the progression of drug resistance. They finally concluded that Cu@Fe NPs can be used for the efficient treatment of infections caused by MRSA.

In an integrative review, Hou et al. deliberated an up-to-date account of antimicrobial photodynamic inactivation as a novel approach that can be used for the treatment of pathogens and may solve the problem of antimicrobial resistance. In this context, fullerene can be used as a photosensitizer for antimicrobial photodynamic inactivation owing to good photostability, O_2_ and reactive oxygen species (ROS) yields, and a broad range of antibacterial activity against Gram-positive and Gram-negative bacteria. The authors reviewed and critically discussed the role of photosensitizers in antimicrobial photodynamic inactivation.

Lastly, it can be concluded that the present Research Topic fortifies the fact that biogenically synthesized nanomaterials have enhanced bioactivity, biocompatibility, and are easy to synthesize in natural conditions. These nanoparticles demonstrate remarkable antimicrobial activities against pathogenic microbes including multidrug resistant (MDR) and extensively drug-resistant (XDR). Such nanomaterials can also be functionalized with polymers. Furthermore, nanoparticles such as fullerene can be used as a photosensitizer for antimicrobial photodynamic inactivation therapy. We hope in the future nanomaterials particularly biogenic will open up new avenues as an alternative treatment strategy. However, more studies are required to address toxicity issues generated by nanomaterials.

## Author contributions

All authors listed have made a substantial, direct, and intellectual contribution to the work and approved it for publication.
